# Supplementary material for: Structure of an Intranucleosomal DNA Loop That Senses DNA Damage during Transcription
Source: Cells. 2022 Aug 28;11(17):2678. doi: 10.3390/cells11172678 (PMC9454427; doi:10.3390/cells11172678)
Supplement: Supplementary file 1 [file cells-11-02678-s001.zip › cells-1864423-supplementary.pdf]

Structure of an Intranucleosomal DNA Loop that Senses DNA Damage during Transcription

Gerasimova N.S., Volokh O.I., Pestov N.A., Armeev G.A., Kirpichnikov M.P., Shaytan A.K., Sokolova O.S., Studitsky V.M.

Supplementary figures S1-S8

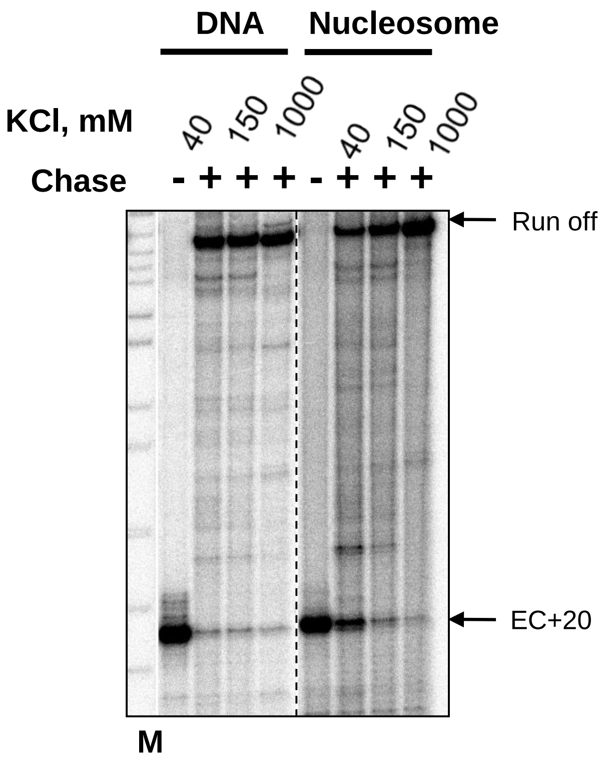

Figure S1. Analysis of stability of stalled EC+20. The RNA-pulse-labeled EC+20 was stalled on DNA or in the nucleosome as described in Figure 1 and then transcribed in the presence of all unlabeled NTPs at different concentrations of KCl. Note nearly quantitative extension of the +24 RNA in the presence of 1M KCl both on DNA and nucleosomes.

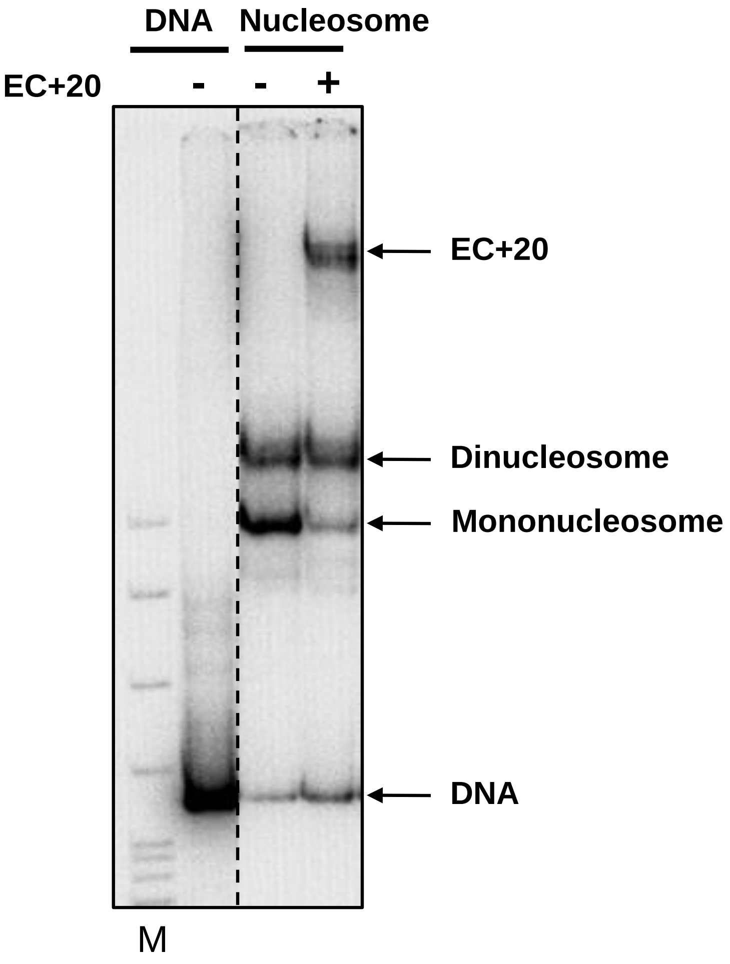

Figure S2. Analysis of stalled EC+20 by non-denaturing PAGE. The DNA-end-labeled EC+20 was stalled on DNA or in the nucleosome as described in Figure 1. Note that EC+20 forms on nucleosomes and contaminating DNA, but not on contaminating dinucleosomes, as was shown previously [68].

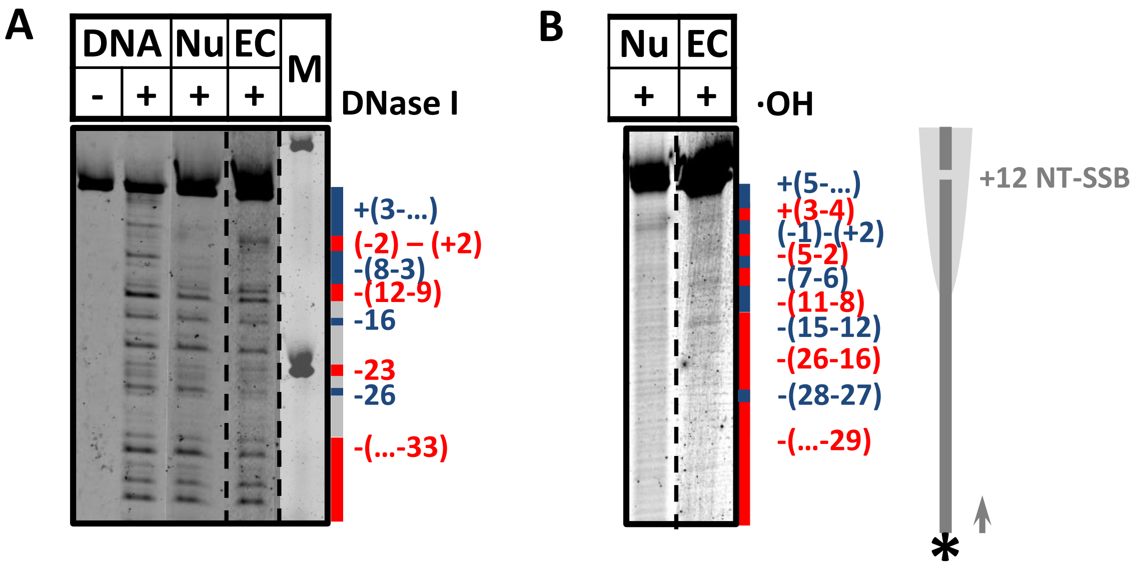

Figure S3. DNase I (A) and hydroxyl radical footprinting (B) of EC+20 arrested in the nucleosome. The EC+20 was arrested on nucleosomes containing end-labeled non-template DNA strand (by FAM or ROX labels, respectively, shown by asterisk) and a single-strand brake at the position +12. EC+20 was formed and footprinted as described in Figure 1. Position of the nucleosome on the template is shown by an oval, the direction of transcription is indicated by arrows. Regions with high sensitivity and protected regions are indicated by red and blue lines, respectively. Since non-template DNA strand contained SSB+12, only relatively short DNA fragments are detectable in the gel.

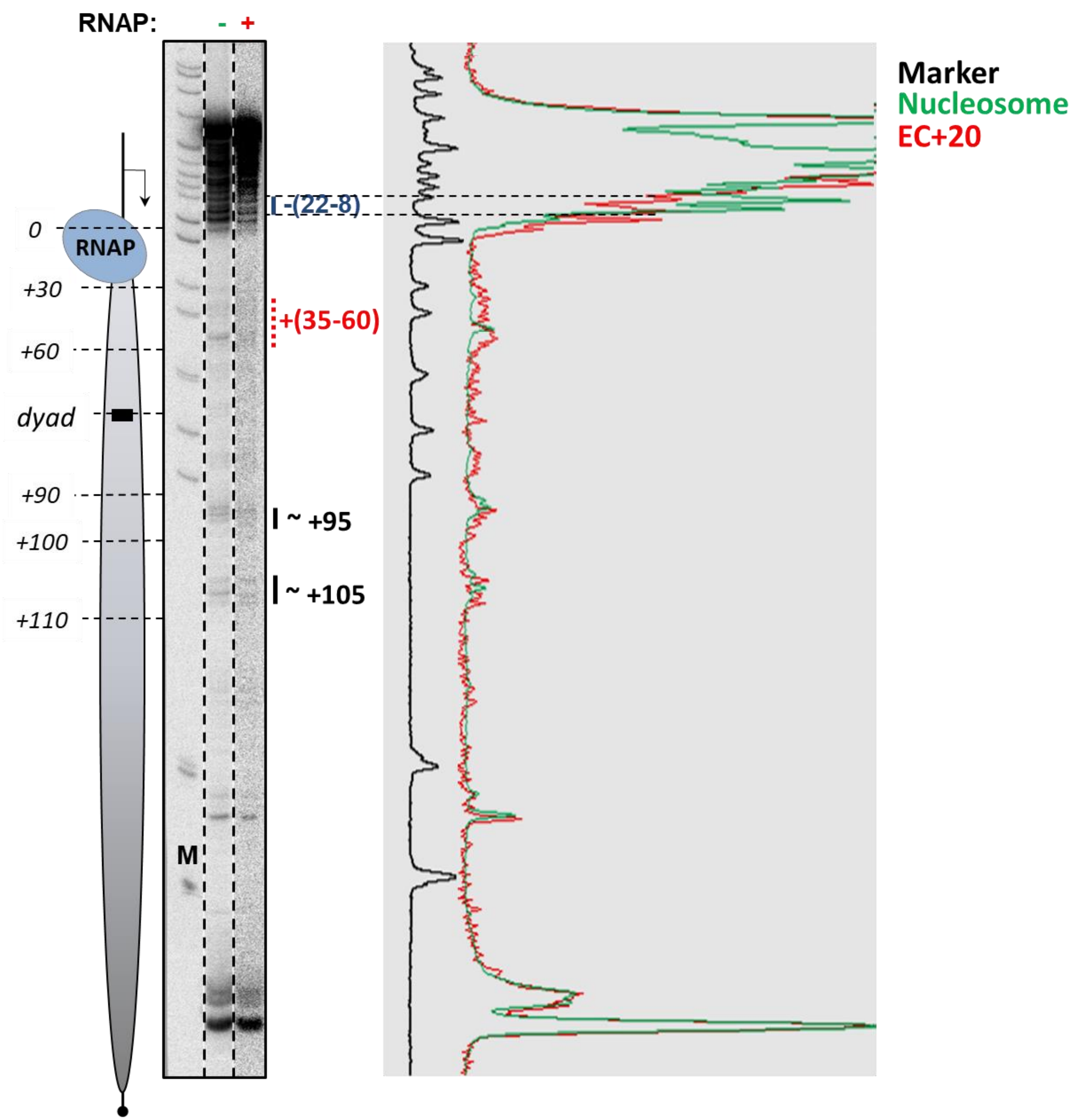

Figure S4. Quantitative analysis of the pattern of DNase I footprinting of EC+20 arrested in the nucleosome containing the +12 nick. Selected lanes on Figure 3 were scanned using OptiQuant software. Band-by-band comparison of the nucleosome and the EC+20 lanes reveals additional protection in promoter part of the DNA (up to ~22 position) and a minor additional sensitivity in the nucleosomal region from position +35 to +60.

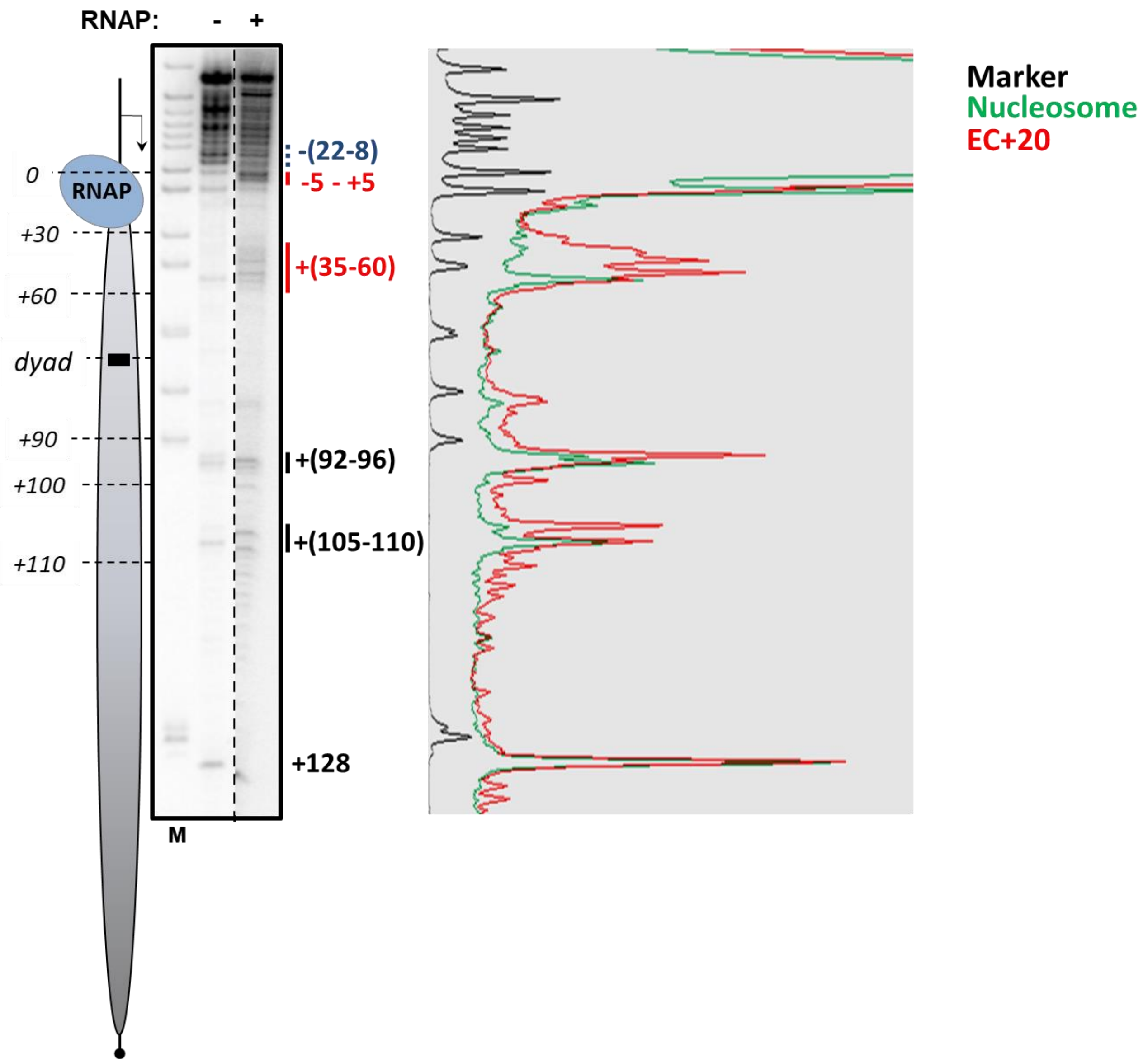

Figure S5. Quantitative analysis of the pattern of DNase I footprinting of EC+20 stalled in intact nucleosome. Selected lanes on Figure 4 were scanned using OptiQuant software. The differences between the lanes are quite pronounced, especially in regions -5 to +5 and +(35-60), because the DNA in the majority of elongation complexes is uncoiled from the histone octamer and is sensitive to DNaseI.

EC+20 with SBB

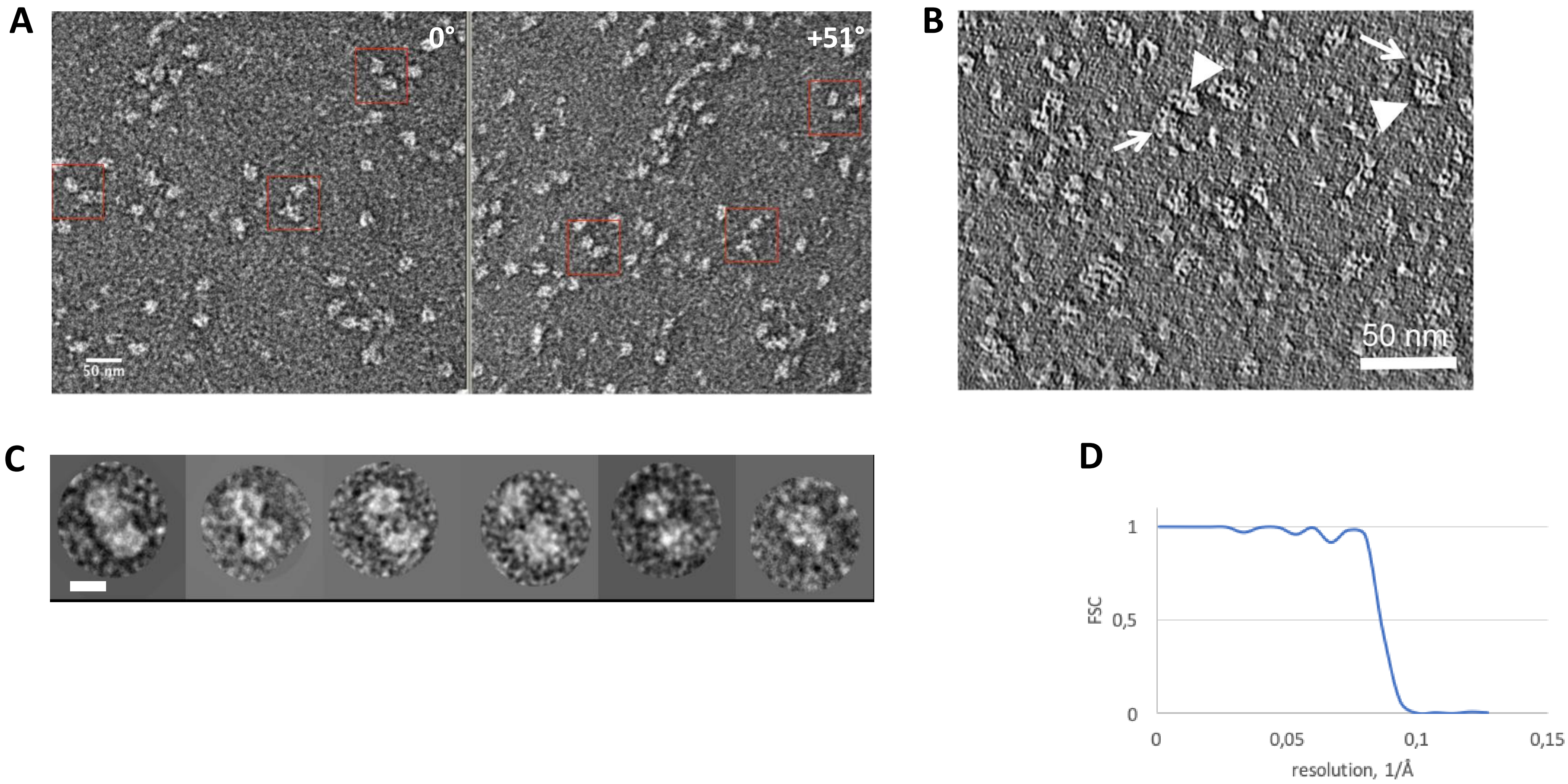

Figure S6. Structure of EC+20 containing +12 SSB: electron microscopy. (A) Related RCT images at 0° (left) and +51° (right) tilt. Red boxes indicate identical complexes viewed from different angles. (B) Tomogram cross-section. Triangles indicate nucleosomes, arrows – RNAP. (C) Representative particles of EC+20 containing +12 SSB. Bar – 10 nm. (D) Fourier shell correlation for 3D-structure of EC+20 with SSB.

EC+20 without SBB

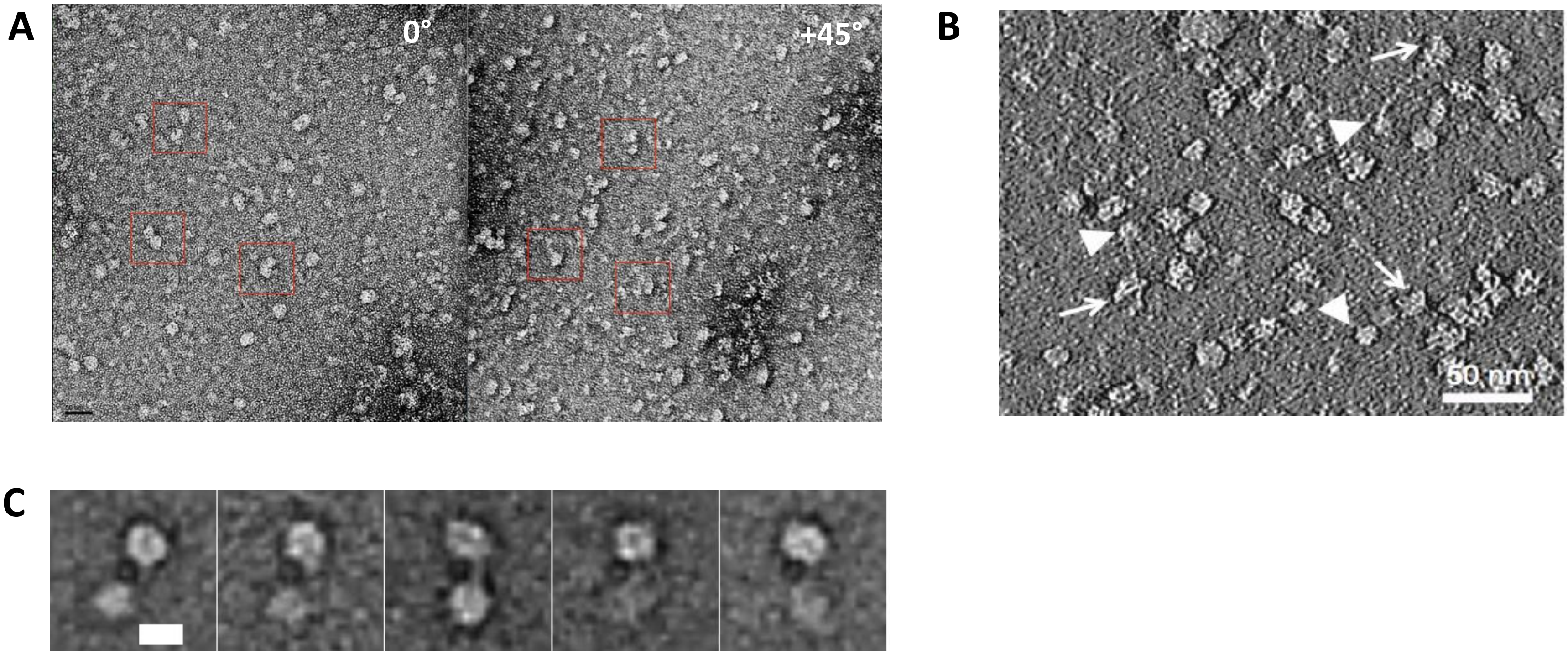

Figure S7. Structure of intact EC+20: electron microscopy. (A) Related RCT images at 0°(left) and +45° (right) tilt. Designations as in Figure S4. (B) Tomogram cross-section. (C) Representative particles of intact EC+20. Bar – 10 nm. RNAP and nucleosome in samples without SSB are considerably further apart, comparing to the samples with SSB (Figure S4).

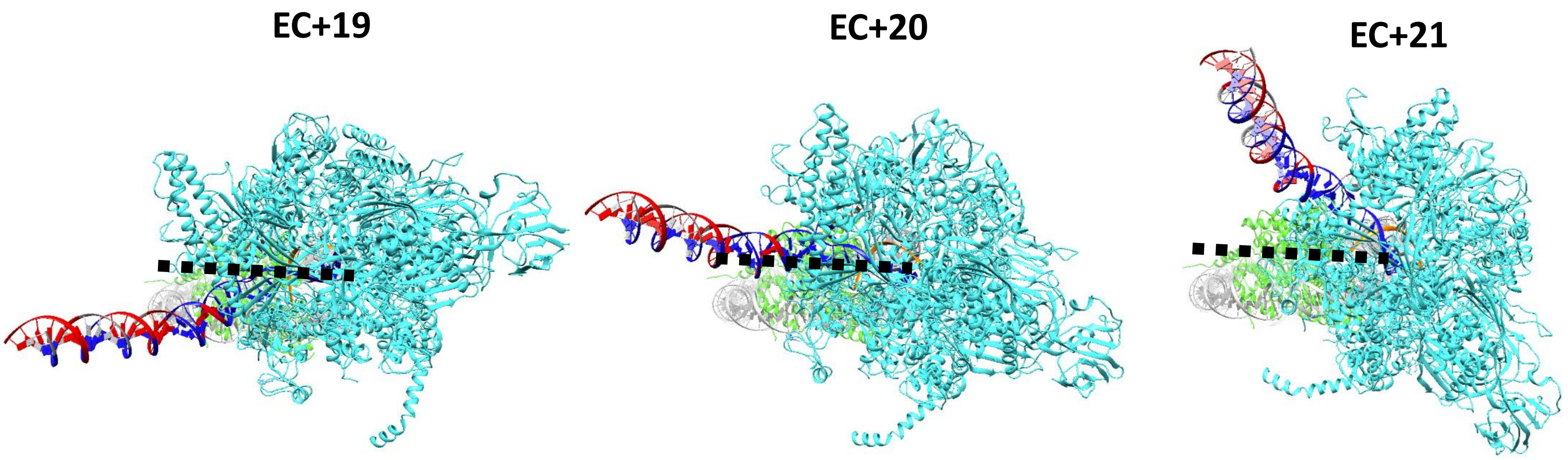

Figure S8. DNA upstream of RNAP is optimally oriented for formation of the i-loop in EC+20. Models of EC+19, EC+20 and EC+21 are shown with the same orientation of the nucleosome. The path of the second supercoil of nucleosomal DNA (that is missing in these complexes because it is uncoiled by RNAP) is shown by dashed lines. Note that the uncoiled DNA is well aligned with the path of the missing DNA supercoil only in EC+20. The color code is the same as in Figure 6.
